# Effect of Adult Weight Gain on Non-Alcoholic Fatty Liver Disease and Its Association with Anthropometric Parameters in the Lean Japanese Population

**DOI:** 10.3390/diagnostics10110863

**Published:** 2020-10-23

**Authors:** Sho Tanaka, Midori Fujishiro, Kentaro Watanabe, Kazuhiro Imatake, Yasuyuki Suzuki, Masanori Abe, Hisamitsu Ishihara, Shigemasa Tani

**Affiliations:** 1 Division of Nephrology, Hypertension and Endocrinology, Department of Internal Medicine, Nihon University School of Medicine, 30-1 Oyaguchi Kami-cho, Itabashi-ku, Tokyo 173-8610, Japan; tanaka.sho@nihon-u.ac.jp (S.T.); abe.masanori@nihon-u.ac.jp (M.A.); 2 Department of Internal Medicine, Nihon University Hospital, 1-6 Surugadai, Kanda, Chiyoda-ku, Tokyo 101-8309, Japan; 3 Division of Diabetes and Metabolic Diseases, Department of Internal Medicine, Nihon University School of Medicine, 30-1 Oyaguchi Kami-cho, Itabashi-ku, Tokyo 173-8610, Japan; watanabe.kentaro@nihon-u.ac.jp (K.W.); ishihara.hisamitsu@nihon-u.ac.jp (H.I.); 4 Department of Health Planning Center, Nihon University Hospital, 1-6 Surugadai, Kanda, Chiyoda-ku, Tokyo 101-8309, Japan; imatake.kazuhiro@nihon-u.ac.jp (K.I.); suzuki.yasuyuki@nihon-u.ac.jp (Y.S.); tani.shigemasa@nihon-u.ac.jp (S.T.); 5 Department of Cardiology, Nihon University Hospital, 1-6 Surugadai, Kanda, Chiyoda-ku, Tokyo 101-8309, Japan; 6 Division of Cardiology, Department of Medicine, Nihon University School of Medicine, 30-1 Oyaguchi Kami-cho, Itabashi-ku, Tokyo 173-8610, Japan

**Keywords:** adult weight gain, body mass index, non-alcoholic fatty liver disease, ultrasonography, waist circumference

## Abstract

Limited data are available on the prevalence of non-alcoholic fatty liver disease (NAFLD) and its association with adult weight gain (AWG) in the lean population. This study aimed to determine the prevalence of NAFLD and to investigate whether AWG is associated with NAFLD in the lean Japanese population. We retrospectively analyzed patients who underwent abdominal ultrasonography as part of the annual health checkup between January 2019 and December 2019. Participants were classified into two groups: those with AWG ≥ 10 kg (AWG group, *n* = 497), and those without gain (non-AWG group, *n* = 3006). To adjust for the confounding effects, we generated 482 pairs using 1:1 propensity score matching. The associations between AWG and NAFLD, anthropometric parameters and NAFLD were investigated using univariate logistic regression analysis. We identified NAFLD in 197 (5.6%) participants. AWG was significantly associated with NAFLD (odds ratio (OR), 1.81; *p* = 0.003). Waist circumference was significantly associated with NAFLD in both the AWG (OR, 1.24; *p* < 0.001) and non-AWG groups (OR, 1.17; *p* < 0.001). The association between body mass index and NAFLD existed in the former group (OR, 1.76; *p* < 0.001), but was not significant in the latter group. AWG is a risk factor for NAFLD even in the lean Japanese population, and associations between anthropometric parameters and NAFLD become stronger if AWG coexists.

## 1. Introduction

Non-alcoholic fatty liver disease (NAFLD) is the most common chronic liver disease characterized by lipid accumulation in hepatocytes in patients without excessive alcohol intake. It encompasses a broad spectrum of liver injury ranging from simple steatosis to steatohepatitis, possibly leading to cirrhosis and hepatocellular carcinoma [1]. The prevalence of NAFLD has been increasing worldwide, with an estimated prevalence rate of approximately 25% in the general population, although it varies according to the region [2]. In Japan, the prevalence rate was reported to be 24.6–29.7% [3,4,5]. A growing epidemic of NAFLD is a significant public health problem because NAFLD is associated with increased risks of overall and liver-related mortality, cardiovascular disease, and impairment of health-related quality of life; although, only lifestyle intervention is the proven treatment [2,6,7,8,9].

NAFLD is closely associated with diverse metabolic comorbidities, including obesity, metabolic syndrome, type 2 diabetes mellitus, dyslipidemia, and hypertension [2,10]. Obesity is recognized as the most significant determinant of NAFLD because the risk of NAFLD proportionally increases with the increase in body mass index (BMI), and most NAFLD patients are obese [2,10,11]. However, some patients with NAFLD are considered lean based on BMI values despite the close association between NAFLD and obesity, and histological features are not milder in lean patients than in their obese counterparts [8,12].

A previous study showed that Asians exhibit higher fat mass despite their lower BMI than whites [13]. Additionally, Asian premenopausal women had significantly larger amounts of visceral adipose tissue even after adjusting for the effect of total body fat than premenopausal European women [14]. These findings suggest that Asians are more prone to be metabolically abnormal than other racial groups. Indeed, a previous cross-sectional study, which compared the clinical manifestations of NAFLD among racial groups in the American population, showed that Asian patients had significantly lower BMI values than other racial groups [15]. Consistently, NAFLD in lean patients, so-called lean NAFLD, is more prevalent among Asian populations than among Western countries [8].

Recently, the World Health Organization recommended that BMI ≥ 23 kg/m^2^ was considered a cutoff for increasing obesity-related health risk and BMI < 23 kg/m^2^ as acceptable categories for Asians considering their racial characteristics [16]. Although a few studies have addressed the prevalence of NAFLD in Japan wherein the patients were lean (BMI < 23 kg/m^2^), the data in Tokyo, the most urbanized area in Japan, remain unclear [17,18]. Furthermore, while an adult weight gain (AWG) is reported to be a risk factor for NAFLD, limited data are available on the association between the AWG and NAFLD in lean Japanese [5]. The present study aimed to investigate the prevalence of NAFLD and the association between NAFLD and AWG in a lean Japanese population.

## 2. Materials and Methods

### 2.1. Participants and Setting

This retrospective cross-sectional study used annual health checkup data from Nihon University Hospital, located in Tokyo, Japan. Participants who had undergone abdominal ultrasonography examination as part of the health checkup between January 2019 and December 2019 were potentially eligible. The exclusion criteria included positive results or missing data for hepatitis B virus surface antigen and/or hepatitis C antibody, alcohol consumption (≥210 g/week for men, ≥140 g/week for women), pregnancy, BMI ≥ 23 kg/m^2^. After the exclusion, participants were classified into two groups according to the presence or absence of AWG ≥ 10 kg: those who had AWG ≥ 10 kg (the AWG group) and those who did not have AWG ≥ 10 kg (the non-AWG group).

### 2.2. Abdominal Ultrasonography

NAFLD was detected on ultrasonography performed in this study, using LOGIQ E9 or LOGIQ S8 (GE Healthcare, Chicago, IL, USA) with a conventional B-mode technique. NAFLD was diagnosed if any of the following ultrasonographic findings were met: bright liver, liver to kidney contrast, deep attenuation, or vascular blurring.

### 2.3. Studied Parameters

BMI was determined by dividing the body weight by the square of height. Waist circumference (WC) was measured in the standing position. Blood pressure (BP) was measured using the oscillometric method, in the sitting position. Information about alcohol consumption; current smoking status; and usage of anti-hypertensive, anti-lipidemic, and anti-diabetic agents; and the presence of weight gain exceeding 10 kg since the age of 20 years was collected using a self-report questionnaire.

Blood samples were collected after an overnight fast. The following laboratory parameters were investigated: high-density lipoprotein cholesterol (HDL-C), low-density lipoprotein cholesterol (LDL-C), triglyceride (TG), glucose, glycated hemoglobin (HbA1c), creatinine, uric acid, aspartate aminotransferase (AST), alanine aminotransferase (ALT), and gamma-glutamyl transpeptidase (G-GTP). Estimated glomerular filtration rate (eGFR) was determined using the following formula: $194\times Creatinine^{-1.094}\times Age^{-0.287}\left(\times 0.739\;if\;female\right)$.

In this study, we defined the coexistence of hypertension as the use of anti-hypertensive agents or systolic BP ≥ 140 mmHg or diastolic BP ≥ 90 mmHg; dyslipidemia as the use of anti-lipidemic agents or HDL-C < 40 mg/dL or LDL-C ≥ 140 mg/dL or TG ≥ 150 mg/dL; diabetes mellitus as use of antidiabetic agents or glucose ≥ 126 mg/dL or HbAc1 ≥ 6.5%.

### 2.4. Statistical Analysis

Data are presented as mean ± standard deviation or as number with percentage. Student’s *t*-test was performed to examine the differences in continuous variables between the two groups. The differences in percentage data were examined using the chi-square test. We used univariate logistic regression analysis to evaluate the associations between AWG ≥ 10 kg and NAFLD and between the anthropometric parameters and NAFLD.

To evaluate the association between NAFLD and AWG, we performed propensity score matching to adjust for background characteristics, including anthropometric parameters between the AWG and non-AWG groups. The propensity score was generated using a logistic regression model using age, sex, BMI, WC, current smoking, anti-hypertensive agent use, systolic BP, anti-lipidemic agent use, HDL-C, LDL-C, TG, anti-diabetic agent use, and HbA1c as confounding variables. One-to-one nearest-neighbor propensity score matching was performed with a caliper width equal to 0.2 times the standard deviation of the logit of the propensity score. After matching, a standardized difference <0.1 was considered adequate covariate balance.

For all statistical analyses, we used EZR version 1.50 (Saitama Medical Center, Jichi Medical University, Saitama, Japan), which is a graphical user interface for R version 3.6.3 (The R Foundation for Statistical Computing, Vienna, Austria). In all analyses, *p* < 0.05 was considered to indicate statistical significance.

### 2.5. Ethics Approval

This study complied with the Declaration of Helsinki and was approved by the Institutional Review Board of Nihon University Hospital (No. 20200404, approved on 9 April 2020) approve code, approved on day month year. Written informed consent requirement was waived because this was a retrospective observational study, and an opt-out recruitment procedure was followed.

## 3. Results

**Figure 1:**
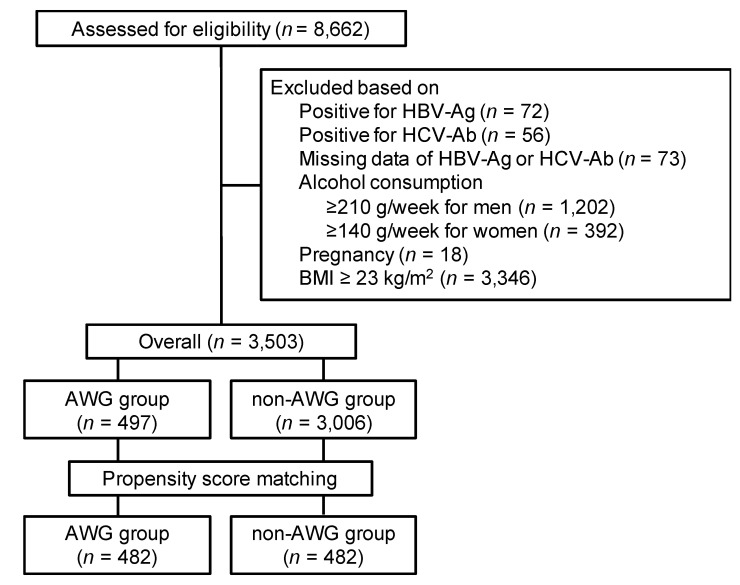
Selection procedure of participants. AWG, adult weight gain; BMI, body mass index; HBs-Ag, hepatitis B virus surface antigen; HCV-Ab, hepatitis C antibody (HCV-Ab).

Figure 1 shows a summary of the participant selection procedure. A total of 8662 participants underwent abdominal ultrasonography during the study period. We excluded 5159 participants based on the exclusion criteria; thus, 3503 participants were eligible for this study. The background characteristics of the participants are listed in Table 1. Of the 3503 eligible participants, 497 had experienced adult weight gain and were categorized into the AWG group; 3006 had not experienced and were categorized into the non-AWG group. Finally, according to the propensity score, we matched 482 (97.0%) participants in the AWG group with 482 participants in the non-AWG group.

Table 2 shows a comparison of the clinical characteristics between the AWG and non-AWG groups. We detected NAFLD in 197 (5.6%) of 3503 participants: 83 in the AWG group and 114 in the non-AWG group. Before matching, the AWG group participants had higher levels of BMI, uric acid levels, and WC and were predominantly male, current smokers, hypertensive, and dyslipidemic than the non-AWG group. Regarding liver status, the AWG group showed higher levels of ALT and G-GTP, and a higher prevalence rate of NAFLD (AWG group vs. non-AWG group; 3.8% vs. 16.7%, *p* < 0.001). After matching, age, sex, anthropometric parameters, smoking status, blood pressure, lipid and glycemic profiles, kidney function, and uric acid level were adequately balanced between the two groups. The difference in G-GTP level was not significant after matching. Meanwhile, the AWG group showed a significantly higher AST level, although it remained within the normal range. Even after matching, NAFLD had a significantly higher prevalence in the AWG group (matched AWG group vs. matched non-AWG group; 9.8% vs. 16.4%, *p* = 0.003).

Table 3 shows the results of univariate logistic regression analysis to assess the relationships between AWG ≥ 10 kg and NAFLD in overall and propensity-matched participants. Although the association between AWG ≥ 10 kg and NAFLD after propensity score adjustment was milder than that before adjustment, AWG ≥ 10 kg remained significantly associated with an increased risk of NAFLD among the propensity-matched cohort (odds ratio (OR), 1.81; 95% confidence interval (CI), [1.23, 2.67]; *p* = 0.003).

**Figure 2:**
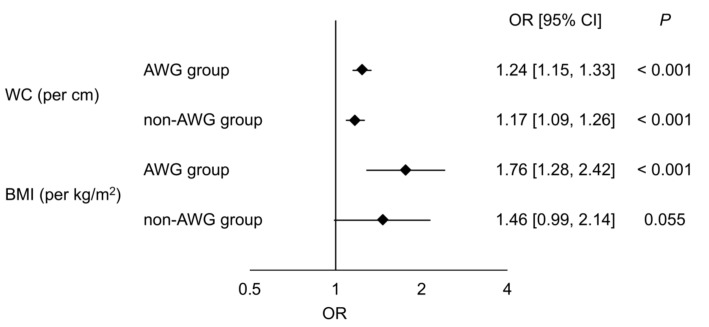
Associations between anthropometric parameters and NAFLD. Odds ratios with 95% confidence intervals for NAFLD evaluated by univariate analysis of waist circumference (per cm) and BMI (per kg/m^2^) between the AWG and non-AWG groups after matching are shown. AWG, adult weight gain; BMI, body mass index; CI, confidence interval; OR, odds ratio; WC, waist circumference.

Figure 2 shows the associations between anthropometric parameters and NAFLD evaluated by univariate logistic regression analysis in the matched AWG and non-AWG groups. WC (per cm) was significantly associated with NAFLD in both the matched AWG (OR, 1.24; 95% CI, [1.15, 1.33]; *p* < 0.001) and non-AWG groups (OR, 1.17; 95% CI, [1.09, 1.26]; *p* < 0.001). We also confirmed a significant association between BMI (per kg/m^2^) and NAFLD in matched participants with AWG ≥ 10 kg (OR, 1.76; 95% CI, [1.28, 2.42]; *p* < 0.001), but the association was not significant in those without AWG ≥ 10 kg (OR, 1.46; 95% CI, [0.99, 2.14]; *p* = 0.055).

## 4. Discussion

We conducted the present study to investigate the association between AWG ≥ 10 kg and NAFLD in the Japanese population being lean, defined as BMI < 23 kg/m^2^. The results showed that AWG ≥ 10 kg was significantly associated with an increased risk of NAFLD even after adjusting for confounding variables, and that WC and BMI were more strongly associated with NAFLD in participants who had experienced AWG ≥ 10 kg than those who had not.

In this study, we detected NAFLD in 5.6% of lean participants, but the rate was relatively low compared with that of previous reports. The prevalence rate of NAFLD in Asian populations with BMI < 23 kg/m^2^ is reported to be diverse, with 30.5% in Taiwan, 14.5% in Bangladesh, 11.1–11.6% in China, 10.3–17.6% in Korea, 8.6% in India, and 4.1% in Malaysia [19,20,21,22,23,24,25,26,27]. In Japan, the prevalence was previously reported in several studies based on annual health checkups, although they slightly differed from each other in the study settings. A multicenter retrospective cross-sectional study conducted at Saga, Hiroshima, and Kochi Prefecture between 2009 and 2010 showed that the prevalence of NAFLD in the population with BMI <23 kg/m^2^ was 10.5% [17]. NAFLD was also surveyed at Gifu Prefecture from 2004 to 2008 and confirmed in 548 of 10,064 (5.4%) non-diabetes patients with BMI < 23 kg/m^2^ [18]. In another cross-sectional study conducted in Kyoto Prefecture between 2012 and 2014, NAFLD was detected in 60 of 456 (13.2%) participants aged 35–75 years, with BMI <23 kg/m^2^ [28]. A post-hoc analysis of the prospective cohort study in 1998 showed that NAFLD was detected in 69 of 984 (7.0%) participants with BMI <23 kg/m^2^ who had not received any medication or had a history of cardiovascular disease [29]. These data and our results suggest that NAFLD is less prevalent in lean adults in Tokyo than in other Asian countries and other regions in Japan, although it should be considered that the heterogeneity of the study settings influences the prevalence rate. It is well known that the prevalence and clinical characteristics of NAFLD vary worldwide, and the rural–urban difference exists even within the same country [30]. As a result that Tokyo is the most urbanized area in Asia, a sedentary lifestyle might promote obesity in NAFLD patients and result in a relatively lower prevalence of lean NAFLD.

An important finding in this study is that AWG ≥ 10 kg is significantly associated with NAFLD in the lean Japanese population. Only limited data are available on this issue. Previously, Nishioji et al. investigated 391 Japanese aged 35–75 years with BMI 18.5–22.9 kg/m^2^ and showed a significant association between AWG ≥ 10 kg and NAFLD (OR, 2.62; 95% CI, [1.32, 5.23]; *p* = 0.0059) after adjusting for the effects of age, sex, *PNPLA3* rs738409 polymorphism, hypertension, type 2 diabetes mellitus, and dyslipidemia [28]. Our result was consistent with that of their report and reinforced the significance of AWG as a risk factor for NAFLD in the lean population.

Additionally, in our present study, the OR of WC (per cm) and BMI (per kg/m^2^) for NAFLD was higher in participants with AWG ≥ 10 kg than in those without, suggesting that AWG strengthens the association between anthropometric indices and NAFLD. As lipid accumulation resulting from positive energy balance exceeds the capacity of peripheral subcutaneous adipose tissue, lipid spillover occurs and leads to visceral and ectopic (i.e., liver, heart, muscle, kidney, and pancreas) fat deposition [31]. A previous cross-sectional study in Japan showed that adults who had larger AWG showed a lower level of adiponectin, which is the biomarker inversely correlated with visceral adipose tissue mass [32,33]. These reports indicate that AWG readily leads to unfavorable fat distribution. Indeed, in a population-based study, AWG was shown to be more strongly associated with hepatic lipid accumulation and visceral adipose tissue excess than subcutaneous adipose tissue in middle-aged adults living in The Western Netherlands [34]. Therefore, adults who experienced AWG are prone to a metabolically abnormal state even if their BMI values are considered lean and increments in WC and BMI can have more detrimental effects on NAFLD development in such populations.

However, this study also has several limitations that should be acknowledged. First, because this was a retrospective cross-sectional study based on an annual health checkup, self-selection bias was not deniable. Second, the generalizability of the study results should be confirmed in another region because this is a single-center study conducted in Tokyo, Japan. The third limitation is that propensity score matching results in well-balanced covariates between the AWG and non-AWG groups, but this method cannot adjust for unknown confounding factors. For instance, iron metabolism previously reported to be associated with lean NAFLD was not evaluated in this study [35]. Additionally, several genetic factors (i.e., *PNPLA3*, *NCAN*, *GCKR*, *LYPLAL1*, and *APOC*) can contribute to NAFLD development, and the G allele of *PNPLA3* rs738409 was shown to be a risk allele in lean Japanese, but genetic factors were not investigated, warranting further studies [8,28].

## 5. Conclusions

The present study showed that AWG is associated with an increased risk of NAFLD in lean Japanese adults. Our findings suggest that anthropometric parameters have greater associations with NAFLD in those who have AWG relative to those who do not have AWG. Physicians should perform a proactive screening for NAFLD in a lean population with a history of AWG.

## Figures and Tables

**Table 1:** Background characteristics of the study participants.

Variables	*n* = 3503
Age, years	49.5 ± 11.3
Male sex, *n* (%)	1587 (45.3%)
BMI, kg/m^2^	20.6 ± 1.7
WC, cm	75.1 ± 6.2
Current smoker, *n* (%)	383 (10.9%)
Hypertension, *n* (%)	476 (13.6%)
Dyslipidemia, *n* (%)	947 (27.0%)
Diabetes mellitus, *n* (%)	123 (3.5%)
AWG, *n* (%)	497 (14.2%)
NAFLD, *n* (%)	197 (5.6%)

Data are presented as mean ± standard deviation, or as number with percentage. AWG, adult weight gain; BMI, body mass index; NAFLD, non-alcoholic fatty liver disease; WC, waist circumference.

**Table 2:** Comparison of clinical and metabolic parameters between participants with and without AWG.

	Before Matching			After Matching		*p*	Std diff
	Non-AWG	AWG	*p*	Non-AWG	AWG		
	(*n* = 3006)	(*n* = 497)		(*n* = 482)	(*n* = 482)		
Age, years	49.4 ± 11.3	49.9 ± 10.9	0.43	49.9 ± 11.4	49.6 ± 10.8	0.64	−0.030
Male sex, *n* (%)	1262 (42.0%)	325 (65.4%)	<0.001	307 (63.7%)	311 (64.5%)	0.84	0.017
BMI, kg/m^2^	20.4 ± 1.7	21.8 ± 0.96	<0.001	21.8 ± 0.9	21.8 ± 1.0	0.47	−0.046
WC, cm	74.2 ± 6.0	80.3 ± 4.5	<0.001	80.0 ± 4.6	80.0 ± 4.3	0.89	0.009
Current smoker, *n* (%)	315 (10.5%)	68 (13.7%)	0.041	70 (14.5%)	67 (13.9%)	0.85	−0.018
Hypertension, *n* (%)	367 (12.2%)	109 (21.9%)	<0.001	96 (19.9%)	104 (21.6%)	0.58	0.041
Systolic BP, mmHg	113.8 ± 12.9	117.3 ± 12.7	<0.001	117.5 ± 13.0	117.3 ± 12.8	0.83	−0.014
Diastolic BP, mmHg	72.3 ± 10.4	75.8 ± 10.4	<0.001	75.7 ± 10.6	75.7 ± 10.5	0.96	−0.003
Dyslipidemia, *n* (%)	746 (24.8%)	201 (40.4%)	<0.001	189 (39.2%)	196 (40.7%)	0.69	0.030
HDL-C, mg/dL	68.6 ± 15.6	60.8 ± 15.6	<0.001	61.2 ± 13.9	60.9 ± 14.0	0.78	−0.018
LDL-C, mg/dL	113.9 ± 27.4	121.9 ± 30.5	<0.001	123.0 ± 27.9	122.2 ± 30.6	0.66	−0.028
TG, mg/dL	73.9 ± 41.1	97.8 ± 57.6	<0.001	96.2 ± 67.1	97.9 ± 58.1	0.66	0.028
Diabetes mellitus, *n* (%)	102 (3.4%)	21 (4.2%)	0.42	14 (2.9%)	19 (3.9%)	0.48	0.057
HbA1c, %	5.67 ± 0.40	5.69 ± 0.38	0.49	5.67 ± 0.39	5.69 ± 0.39	0.37	0.058
eGFR, mL/min/1.73 m^2^	76.6 ± 13.2	76.1 ± 12.6	0.44	76.2 ± 12.8	76.3 ± 12.5	0.87	0.011
Uric acid, mg/dL	4.75 ± 1.22	5.58 ± 1.29	<0.001	5.49 ± 1.24	5.56 ± 1.30	0.41	0.054
AST, U/L	20.7 ± 7.0	21.3 ± 9.0	0.14	21.1 ± 7.2	21.2 ± 9.1	0.76	0.020
ALT, U/L	17.6 ± 9.6	21.5 ± 12.1	<0.001	19.7 ± 11.2	21.5 ± 12.2	0.018	0.152
G-GTP, U/L	24.8 ± 26.0	36.2 ± 76.4	<0.001	30.2 ± 36.9	36.0 ± 77.3	0.14	0.096
NAFLD, *n* (%)	114 (3.8%)	83 (16.7%)	<0.001	47 (9.8%)	79 (16.4%)	0.003	0.198

Data are presented as mean ± standard deviation, or as number with percentage. ALT, alanine aminotransferase; AST, aspartate aminotransferase; AWG, adult weight gain; BMI, body mass index; BP, blood pressure; eGFR, estimated glomerular filtration rate; G-GTP, gamma-glutamyl transpeptidase; HbA1c, glycated hemoglobin; HDL-C, high-density lipoprotein cholesterol; LDL-C, low-density lipoprotein cholesterol; NAFLD, non-alcoholic fatty liver disease; Std diff, standardized difference; TG, triglyceride; WC, waist circumference.

**Table 3:** Univariate logistic regression analysis using NAFLD as the dependent variable.

	Before Matching (*n* = 3503)		After Matching (*n* = 964)	
	OR [95% CI]	*p*	OR [95% CI]	*p*
AWG	5.09 [3.76, 6.87]	<0.001	1.81 [1.23, 2.67]	0.003

AWG, adult weight gain; CI, confidence interval; NAFLD non-alcoholic fatty liver disease; OR, odds ratio.
